# Analysis of the correlations between insomnia and mental health during the COVID-19 pandemic in Germany

**DOI:** 10.1007/s11818-022-00347-7

**Published:** 2022-05-16

**Authors:** Ying Huang, Ingo Fietze, Thomas Penzel

**Affiliations:** Interdisciplinary Sleep Medicine Center, Charité—Universitätsmedizin Berlin, Chariteplatz 1, 10117 Berlin, Germany

**Keywords:** Anxiety, Depression, Sleep, Coronavirus, Quality of life, Angst, Depression, Schlaf, Coronavirus, Lebensqualität

## Abstract

**Objective:**

This study evaluates correlations between insomnia and mental health during the COVID-19 pandemic in Germany.

**Methods:**

The internet-based International COVID-19 Sleep Study (ICOSS) questionnaire including sociodemographic questions as well as sleep- and emotion-related scales was distributed in Germany during the COVID-19 pandemic from May 1 to September 30, 2020. Insomnia and mental state were assessed using the Insomnia Severity Index (ISI), Patient Health Questionnaire (PHQ), Generalized Anxiety Disorder (GAD-2), and visual analogue scale. Qualitative analyses of demographic characteristics were performed and correlation analyses of the variables calculated.

**Results:**

A total of 1103 individuals participated and 858 valid questionnaires (70.61% females) were obtained. Mean age and body mass index were 41.97 ± 12.9 years and 26 ± 5.9 kg/m^2^, respectively. Most participants were married (*n* = 486, 56.6%), living in the city (*n* = 646, 75.3%), and white (*n* = 442, 51.5%). The prevalence of insomnia, anxiety, and depression was 19.5% (ISI > 7), 6.6% (GAD-2 > 3), and 4.8% (PHQ-2 > 3), respectively. Compared to the insomnia group, the mean and median ISI, PHQ‑4, PHQ‑2, and GAD‑2 scores of the non-insomnia group were lower, while their mean and median quality of life and quality of health scores were significantly higher (*P* < 0.05). Pearson correlation analysis showed a positive correlation between the ISI and PHQ‑2 (*r* = 0.521, *P* < 0.001), GAD‑2 (*r* = 0.483, *P* < 0.001), and PHQ‑4 scores (*r* = 0.562, *P* < 0.001); however, the ISI score negatively correlated with the quality of life (*r* = −0.490, *P* < 0.001) and quality of health scores (*r* = −0.437, *P* < 0.001).

**Conclusion:**

Insomnia, anxiety, and depression were very prevalent during the pandemic. Anxiety and depression were more severe in the insomnia than in the non-insomnia group, and insomnia and mental health are closely related.

## Abstract (de)

**Zielsetzung:**

Diese Studie untersucht die Zusammenhänge zwischen Schlaflosigkeit und psychischen Gesundheitsfaktoren während der COVID-19-Pandemie in Deutschland.

**Methoden:**

Der internetbasierte Fragebogen der Internationalen COVID-19-Schlafstudie (ICOSS), der auch soziodemographische Fragen sowie schlaf- und emotionsbezogene Skalen enthielt, wurde während der COVID-19-Pandemie vom 1. Mai bis 30. September 2020 in Deutschland verteilt. Schlaflosigkeit und psychischer Zustand wurden mit dem Insomnia Severity Index (ISI), dem Patient Health Questionnaire (PHQ), der Generalized Anxiety Disorder (GAD-2) und der visuellen Analogskala erfasst. Qualitative Analysen der demographischen Merkmale wurden durchgeführt und Korrelationsanalysen der Variablen berechnet.

**Ergebnisse:**

Insgesamt nahmen 1103 Personen an der Studie teil und 858 gültige Fragebögen (70,61 % Frauen) wurden ausgefüllt. Das Durchschnittsalter der Teilnehmer betrug 41,97 ± 12,9 Jahre, der durchschnittliche Body-Mass-Index 26 ± 5,9 kg/m^2^. Die meisten Teilnehmer waren verheiratet (*n* = 486, 56,6 %), lebten in der Stadt (*n* = 646, 75,3 %) und waren weiß (*n* = 442, 51,5 %). Unter den Teilnehmern lag die Prävalenz von Schlaflosigkeit, Angst und Depression bei 19,5 % (ISI > 7), 6,6 % (GAD-2 > 3) bzw. 4,8 % (PHQ-2 > 3). Im Vergleich zur Insomniegruppe waren die Mittelwerte und Mediane der ISI-, PHQ-4-, PHQ-2- und GAD-2-Scores der Nichtinsomniegruppe niedriger, während deren Lebensqualitäts- und Gesundheitsqualitätsscores höhere, statistisch signifikante (*p* < 0,05) Mittelwerte und Mediane aufwiesen. Die Pearson-Korrelationsanalyse zeigte eine positive Korrelation zwischen dem ISI- und PHQ-2-Score (r = 0,521, *p* < 0,001) sowie dem GAD-2- (r = 0,483, *p* < 0,001) und PHQ-4-Score (r = 0,562, *p* < 0,001); der ISI-Score war jedoch negativ korreliert mit dem Lebensqualitäts- (r = −0,490, *p* < 0,001) und Gesundheitsqualitätsscore (r = −0,437, *p* < 0,001).

**Schlussfolgerung:**

Schlaflosigkeit, Angstzustände und Depression waren während der Pandemie weit verbreitet. Angstzustände und Depression waren in der Insomniegruppe stärker ausgeprägt als in der Nichtinsomniegruppe; Schlaflosigkeit und psychische Gesundheit sind eng miteinander verbunden.

## Introduction

Coronavirus disease 2019 (COVID-19) is a respiratory disease that is currently considered a global threat for its extreme infectivity and high transmission rate [1]. On March 11, 2020, the World Health Organization (WHO) declared COVID-19 as a pandemic, therefore becoming the first pandemic of this century [2].

The COVID-19 pandemic has not only had a significant impact on individuals, industries, the economy, and health systems, but has also caused unimaginable collateral damage. Since the beginning of the pandemic, a mental health crisis has also followed, and symptoms of anxiety and depression have occurred more frequently [3]. As of writing this article, there have been more than 200 million confirmed cases and over 4.1 million deaths in more than 200 countries, including Germany [4]. In Germany, the first COVID-19 patient was confirmed on January 27, 2020, and the infection quickly spread nationwide [5]. The first wave of COVID-19 in Germany occurred throughout February 2020. On August 19, 2021, the Robert Koch Institute (RKI) demonstrated Germany had started the fourth wave of COVID-19, especially among young people [6]. To reduce the spread of COVID-19, the RKI, the German Federal Government, and the state governments focused on and implemented precautionary measures such as the mandatory use of face masks in public places, extensive COVID-19 screening tests, social distancing, self-quarantine, vaccination, and other measures [7]. However, the incidence of COVID-19 continued to rise and fall, and as of September 6, 2021, the daily number of new confirmed cases was around 5000 [8].

Several studies published on previous epidemics (e.g., severe acute respiratory syndrome, SARS; Ebola) have reported that the massive outbreak increased cases of depression and anxiety among the general public, therefore highlighting the importance of psychiatric assessment and early intervention to decrease susceptibility to mental disorders during an epidemic [9, 10]. COVID-19 is anticipated to have a greater impact on mental health than the previously mentioned epidemics [11].

Insomnia is a clinical disorder characterized by difficulty in falling asleep, difficulty in sleep maintenance, early awakening, and nonrestorative sleep. It exists widely in the general population, and studies from Norway, Britain, and Germany show that the prevalence of insomnia has increased to about 10% of the population in recent years [12–14]. At present, the subjective tool for clinical evaluation of insomnia and sleep quality is the Insomnia Severity Index (ISI) scale, which has the advantages of simplicity, easy operation, and low time consumption.

The International COVID-19 Sleep Study (ICOSS) is an international collaboration involving a group of sleep experts worldwide who focus on describing the nature and rates of various sleep and circadian rhythm symptoms [15]. Previous studies that made use of the ICOSS questionnaire have shown that participants at high risk of obstructive sleep apnea had increased odds of having COVID-19 and were two times more likely to be hospitalized or treated in intensive care units [16]. Additionally, it was also shown in a study by Morin that insomnia, anxiety, and depression were prevalent during the first wave of the COVID-19 pandemic [17].

However, to the best of our knowledge, the associations between insomnia and mental health-related factors during the COVID-19 pandemic have not yet been explored in Germany. Hence, as part of an international collaboration, our study aimed to use a cross-sectional design to evaluate correlations between insomnia and mental health-related factors during COVID-19 in Germany.

## Methods

### Questionnaire

The ICOSS questionnaire is composed of 50 questions with a total of 106 items, including sociodemographic information such as gender, age, height, weight, marital status, living area, educational level, race, occupation, infection with COVID-19, economic impact, and smoking and drinking habits. Moreover, the survey also contains sleep-related and emotion-related scales, including the ISI [18], the Patient Health Questionnaire (PHQ) for Depression and Anxiety [19], the WHO-Five Well-Being Index (WHO-5) [20], and the STOP questionnaire (snoring, tiredness, observed apnea, and high blood pressure) [21].

#### Insomnia

The ISI scale was specifically designed to evaluate the degree of insomnia. This questionnaire contains seven items, and each item was divided into five levels with 0–4 points per item [18]. The higher the score, the more severe the insomnia. A score of 0–7 points indicates the absence of insomnia; 8–14 points, mild insomnia; 15–21 points, moderate insomnia; and 22–28 points, severe insomnia. Previous reports have shown the reliability and validity of the ISI scale in evaluating insomnia [22]. Similarly, this study reports a relatively high internal consistency of the ISI scale (α = 0.873).

#### Depression and anxiety

Mental health was assessed using the anxiety and depression scale (PHQ-4), which is a shorter version of the PHQ‑9 scale [23]. This scale includes the first two questions of the PHQ‑2 depression module and the first two questions of the Generalized Anxiety Disorder‑2 (GAD-2) module. Several studies have demonstrated the reliability of PHQ‑2 and GAD‑2 as screening tools [24–27]. The possible answers for this section are “not at all,” “on certain days,” “on more than half of the days,” and “almost every day,” with values of 0 to 3 assigned to each. The PHQ‑4 scale is evaluated as a sum of the four items, which is a value between 0 and 12. For PHQ‑2 and GAD‑2, a total score > 3 indicates a clinically relevant depression and anxiety disorder [25, 28, 29].

#### Quality of life and quality of health

Quality of life and quality of health were measured using a 0–100 visual analogue scale.

### Participants

From May to September 2020, the questionnaires were collected online through the REDCap software (Version 8.0, Vanderbilt, Nashville, TN, USA) or by distributing physical questionnaires. A total of 1150 subjects completed the questionnaire. We excluded those under the age of 18 years and those with incomplete target data. All participants gave informed consent prior to the survey. The study was conducted in accordance with the Declaration of Helsinki and approved by the local ethics committee of the Charite University Medicine Berlin (reference number EA1/161/20).

### Statistical analysis

Data processing and analysis was performed using SPSS version 23.0 for Mac (IBM Corp., Armonk, NY, USA). The data were tested for normality using the Shapiro–Wilk test. Normal data were analyzed using Student’s *t*-test of two independent samples and were described as mean ± standard deviation (x ± s). Non-normal data were described by the median and were analyzed by Mann–Whitney U test. Categorical data were presented as a frequency (*N*) and percentage (%), and statistical significance was evaluated using the chi-square or Fisher’s exact test. The correlation between insomnia and mental health-related factors was detected using Pearson correlation. Binary logistic regression was then adopted to explore the risk factors of insomnia and mental health-related problems, and the results were described as the odds ratio (OR) with a confidence interval (CI) of 95%. All data were analyzed using a two-tailed test and a significance level set at *P* < 0.05.

## Results

### Comparison of sociodemographic characteristics

The 1103 questionnaires were distributed uniformly, and 858 valid questionnaires (70.61% females) were obtained after excluding those with incomplete scale tests and questionnaires with missing general data. Table 1 presents the sociodemographic characteristics of the participants. Results show that the participants were mostly females (*n* = 661, 77%) and that the mean age and mean body mass index were 41.97 ± 12.9 years and 26 ± 5.9 kg/m^2^, respectively. Most of the participants were married (*n* = 486, 56.6%), living in the city (*n* = 646, 75.3%), employed (*n* = 692, 80.7%), and white (*n* = 442, 51.5%). Among the participants, 15 (1.7%) were diagnosed with COVID-19, 123 participants (14.3%) suffered from “somewhat economic impact,” and 37 (4.3%) suffered from “severe economic impact”; 86 participants (10.0%) were smokers and 301 participants (35.1%) suffered from stress.

**Table 1:** Characteristics of the study population (*N* = 858)

Variables		*N*	%
Gender	Male	197	22.9
	Female	661	77.0
Age (x ± s, year)	–	41.97 ± 12.9	–
BMI (x ± s, kg/m^2^)	–	26 ± 5.9	–
Marital status	Single	331	38.6
	Married	486	56.6
	Others	41	4.8
Living areas	City	646	75.3
	Country	212	24.7
Education level	Less than bachelors	536	62.5
	Bachelors or higher	322	37.6
Ethnicity	White	442	51.5
	Asian	70	8.2
	African	20	2.3
	Hispanic	25	2.9
	Others	301	35.1
Employment status	Student	72	8.4
	Employed	692	80.7
	Unemployed	94	11
COVID-19	No	746	86.9
	Yes	15	1.7
	Don’t know	97	11.3
Financial suffering	None to little	698	81.4
	Somewhat	123	14.3
	Much to severely	37	4.3
Smoking	No	772	90.0
	Yes	86	10.0
Stress	None to rarely	557	64.9
	Some extent to very much	301	35.1
Quality of sleep (x ± s, score)		83.21 ± 13.0	–
Quality of health (x ± s, score)		83.6 ± 12.9	–

Values are presented as number (percentage) or the mean ± standard deviation (x ± s)

*BMI* body mass index

According to their ISI scores, participants were assigned to the non-insomnia group (691 cases) or the insomnia group (167 cases). Table 2 shows the demographic data and scale information of the insomnia and non-insomnia groups in Germany. Compared to females of the insomnia group, males of the same group were linked to a greater prevalence of insomnia (36.6 versus 14.5%), and there are significant differences in gender (χ^2^ = 46.95, *P* < 0.05) compared with the non-insomnia group. In terms of marital status, the prevalence of insomnia was 18.1% in the single participants, 17.3% in the married participants, and 56.1% in those with other marital statuses. Similarly, there are significant differences in marital status (χ^2^ = 36.95, *P* < 0.05) compared with the non-insomnia group. There were also significant differences in education level (χ^2^ = 33.14, *P* < 0.05), ethnicity (χ^2^= 11.57, *P* < 0.05), employment status (χ^2^ = 82.31, *P* < 0.05), financial suffering (χ^2^ = 45.75, *P* < 0.05), smoking status (χ^2^ = 10.46, *P* < 0.05), and stress levels (χ^2^ = 113.44, *P* < 0.05) between the two groups, but the results showed no significant correlations between insomnia and living areas (χ^2^ = 0.02, *P* > 0.05) or COVID-19 groups (χ^2^ = 3.99, *P* > 0.05).

**Table 2:** Comparison of sociodemographic characteristics between non-insomnia and insomnia groups

Variables		Non-insomnia	Insomnia	χ^2^- or *t*-text value
		(*N* = 691)	(*N* = 167)	
Gender	Male	123(63.4)	71(36.6)	46.95*
	Female	568(85.5)	96(14.5)	
Age (x ± s, year)	–	40.42 ± 11.7	46.52 ± 15.1	−5.28*
BMI (x ± s, kg/m^2^)	–	24.73 ± 4.5	27.56 ± 7.2	–
Marital status	Single	271(81.9)	60(18.1)	36.95*
	Married	402(82.7)	84(17.3)	
	Other	18(43.9)	23(56.1)	
Living areas	City	521(80.7)	125(19.3)	0.022
	Country	170(80.2)	42(19.8)	
Education level	Less than bachelor’s	464(86.6)	72(13.4)	33.14*
	Bachelor’s or higher	227(70.5)	95(29.5)	
Ethnicity	White	375(84.8)	67(15.2)	11.57*
	Asian	54(77.1)	16(22.9)	
	African	14(70)	6(30.0)	
	Hispanic	20(80)	5(20.0)	
	Others	228(75.7)	73(24.3)	
Employment status	Student	56(77.8)	16(22.2)	82.31*
	Employed	591(85.5)	100(14.5)	
	Unemployed	44(46.3)	51(53.7)	
COVID-19	No	593(79.5)	153(20.5)	3.99
	Yes	13(86.7)	2(13.3)	
	Don’t know	85(87.6)	12(12.4)	
Financial suffering	None to little	572(81.9)	126(18.1)	45.75*
	Somewhat	105(85.4)	18(14.6)	
	Much to severely	14(37.8)	23(62.2)	
Smoking	No	633(82.0)	139(18)	10.46*
	Yes	58(67.4)	28(32.6)	
Stress	None to rarely	483(86.7)	74(13.3)	113.443*
	Some extent	194(78.2)	54(21.8)	
	Much to severely	14(22.9)	39(77.1)	
Quality of life (x ± s, score)		86.01 ± 8.30	71.37 ± 20.35	14.45*
Quality of health (x ± s, score)		85.92 ± 8.76	73.89 ± 18.74	12.11*

Values are presented as number (percentage) or the mean ± standard deviation (x ± s)

*BMI* body mass index

*p < 0.05

### Comparison of sleep- and emotion-related scales

Among the participants, 19.5% had symptoms of insomnia according to the ISI score (> 7), 6.6% had symptoms of anxiety according to the GAD‑2 score (> 3), and 4.8% had symptoms of depression according to the PHQ‑2 score (> 3) (Table 3).

**Table 3:** Demographics of the scores

Item	Score	*N*	%
*ISI*			
–	≤ 7	691	80.5
	> 7	167	19.5
*PHQ‑4*			
–	≤ 3	738	82.6
	> 3	156	17.5
*GAD‑2*			
–	≤ 3	835	93.4
	> 3	59	6.6
*PHQ‑2*			
–	≤ 3	851	95.2
	> 3	43	4.8

Values are presented as number (*N*) and percentage (%)

*ISI* Insomnia Severity Index, *PHQ* the Patient Health Questionnaire for Depression and Anxiety, *GAD* Generalized Anxiety Assessment module

Comparing the scores of sleep- and emotion-related scales between the two groups, the ISI score, PHQ‑4 score, PHQ‑2 score, and GAD‑2 score of the non-insomnia group had significantly lower mean scores (*P* < 0.001). On the other hand, the non-insomnia group had significantly higher mean scores in quality of life and quality of health compared with the insomnia group (*P* < 0.001, Table 4).

**Table 4:** Comparison of ISI, PHQ‑4, PHQ‑2, GAD‑2, quality of life, and quality of health scores between the insomnia group and non-insomnia groups

Item	Non-insomnia	Insomnia	*Z*	*P*-value
	x ± s (median)	x ± s (median)		
ISI	4.03 ± 1.2(4)	12.64 ± 4.94(11)	*−20.62*	< 0.001
PHQ–4	5.823 ± 0.96(6)	7.44 ± 2.66(7)	−8.361	< 0.001
PHQ‑2	0.94 ± 0.56(1)	1.7 ± 1.42(2)	−9	< 0.001
GAD‑2	0.88 ± 0.6(1)	1.6 ± 1.4(1)	−6.385	< 0.001
Quality of life	86.01 ± 8.3(88.0)	71.37 ± 20.34(75)	−8.72	< 0.001
Quality of health	85.92 ± 8.758(89)	73.89 ± 18.74(78)	−7.32	< 0.001

*ISI* Insomnia Severity Index, *PHQ* the Patient Health Questionnaire for Depression and Anxiety, *GAD* Generalized Anxiety Assessment module, *x* *±* *s* mean ± standard deviation

### Correlations between sleep- and emotion-related scales

The correlations between insomnia (ISI scale), anxiety (GAD‑2 scale), and depression (PHQ‑2 scale) were further analyzed. Pearson correlation analysis showed that there is a positive correlation between the ISI score and the PHQ‑2 score (*r* = 0.521, *P* < 0.01), GAD‑2 score (*r* = 0.483, *P* < 0.01), and PHQ‑4 score (*r* = 0.562, *P* < 0.01). Conversely, the ISI score was negatively correlated with the quality of life score (*r* = −0.490, *P* < 0.01) and the quality of health score (*r* = −0.437, *P* < 0.01). The detailed results are shown in Table 5.

**Table 5:** Correlation analysis of ISI scores with PHQ‑2, GAD‑2, stress, quality of life and quality of health

Scales	1. ISI	2. PHQ‑2	3. GAD‑2	4. PHQ‑4	5. Quality of life	6. Quality of health
1. ISI	–	–	–	–	–	–
2. PHQ‑2	0.521**	–	–	–	–	–
3. GAD‑2	0.483**	0.616**	–	–	–	–
4. PHQ‑4	0.562**	0.900**	0.898**	–	–	–
5. Quality of Life	−0.490**	−0.452**	−0.448**	−0.505**	–	–
6. Quality of Health	−0.437**	−0.311**	−0.263**	−0.323**	0.713**	–

*ISI* Insomnia Severity Index, *PHQ* the Patient Health Questionnaire for Depression and Anxiety, *GAD* generalized anxiety assessment module

**p < 0.01

### Logistic regression analysis of insomnia, anxiety, and depression

The binary logistic regression method was used for multivariate analysis. The group of participants with ISI score > 7, PHQ‑2 score > 3, and GDA‑2 score > 3 were divided into normal and symptomatic groups as dependent variables, and three logistic regression models were established for each. On the other hand, gender, marital status, living areas, educational level, ethnicity, employment status, financial suffering, smoking, and stress were considered as independent variables, with the last category used as the reference. Table 6 describes the results of the multivariate logistic regression analysis. Findings showed that compared with females, males (OR = 2.59, 95% CI 1.789–3.759, *P* < 0.001) had a higher risk for developing clinical insomnia. Compared with living in the country, living in the city (OR = 2.40, 95% CI 1.064–5.413, *P* = 0.035) was observed to be a potential risk factor for depression. Lastly, Asian ethnicity (OR = 3.32, 95% CI 1.056–10.435, *P* = 0.04) and no smoking (OR = 5.99, 95% CI 1.947–18.377, *P* = 0.002) were potential risk factors for anxiety.

**Table 6:** Logistic regression analysis of insomnia, anxiety, and depression in German persons

Item	Variable	B	SE	*P*-value	OR	Lower limit of 95% CI	Upper limit of 95% CI
*ISI* *>* *7*							
Gender	Man	0.953	0.189	0	2.593	1.789	3.759
Marital status	Single	−0.816	0.363	0.025	0.442	0.217	0.901
	Married	−0.832	0.351	0.018	0.435	0.219	0.866
Financial suffering	None to little	−1.133	0.409	0.006	0.322	0.145	0.718
	Somewhat	−0.933	0.451	0.038	0.393	0.163	0.951
Employment status	Student	−1.148	0.373	0.002	0.317	0.153	0.66
*PHQ‑2* *>* *3*							
Living areas	City	0.875	0.415	0.035	2.4	1.064	5.413
Education level	Less than bachelor’s	−0.779	0.311	0.012	0.459	0.249	0.844
Stress	None to rarely	−3.439	0.425	< 0.001	0.032	0.014	0.074
	Some extent	−2.535	0.423	< 0.001	0.079	0.035	0.182
Employment status	Employed	−0.972	0.419	0.02	0.378	0.166	0.86
*GDA‑2* *>* *3*							
Ethnicity	Asian	1.2	0.584	0.04	3.319	1.056	10.435
Smoking	No	1.791	0.574	0.002	5.998	1.947	18.477
Stress	None to rarely	−2.393	0.488	< 0.001	0.091	0.035	0.238
	Some extent	−1.515	0.482	0.002	0.22	0.085	0.565
Employment status	Student	−1.327	0.656	0.043	0.265	0.073	0.96
	Employed	−0.998	0.44	0.023	0.369	0.155	0.874

*ISI* Insomnia Severity Index, *PHQ* the Patient Health Questionnaire for Depression and Anxiety, *GAD* Generalized Anxiety Assessment module, *95% CI* 95% confidence interval, *OR* odds ratio

## Discussion

Similar to previous epidemics, the COVID-19 pandemic was foreseen to greatly impact mental health worldwide. In addition, factors such as social environment and one’s health may also contribute to the instability of emotional state, which is one of the susceptibility factors for insomnia [30]. This study conducted an ICOSS survey among the general population in Germany during the COVID-19 outbreak. We utilized different scales to assess overall mental health and the susceptibility of the respondents to insomnia. Moreover, relevant factors were examined to determine the associations between insomnia and mental health-related factors.

The ISI scale has long been proven to have high confidence and validity. The contents of this scale are based on the diagnostic criteria for insomnia in sleep disorders, as stated in the Diagnostic and Statistical Manual of Mental Disorders (DSM, 5th ed) [18, 31]. On the other hand, the PHQ‑2 and GAD‑2 scales are widely used in screening for anxiety and depression [23–27]. In this study, the prevalence of insomnia, anxiety, and depression was 19.5% (ISI > 7), 6.6% (GAD-2 > 3), and 4.8% (PHQ-2 > 3), respectively, all of which are a substantial increase compared with the pre-pandemic situation. Before the outbreak, the prevalence of insomnia in Germany was 5.7%, with females two times more likely to develop insomnia than males [32]. A recent meta-analysis summarizing the prevalence of anxiety and depression in the general population during the COVID-19 pandemic reported prevalence rates of 31.9% (95% CI, 27.5–36.7) and 33.7% (95% CI, 27.5–40.6), respectively [33]. As seen in the demographic characteristics (Table 2), males are more likely to develop insomnia than females. In this study, the proportion of males with insomnia was higher than that of females. This is inconsistent with several studies showing that severe insomnia is more prevalent in females [34]. Those who are living alone after divorce or separation, are unemployed, have a bachelor’s degree or higher education level, have higher stress levels, have high economic losses, and with no prior COVID-19 infection comprised the majority of the insomnia group. Similarly, several studies have also found that severe insomnia was more prevalent among those who are unemployed, those living alone after divorce or separation, and those living in large cities [35–37].

However, this study is of cross-sectional design, and no causal relationship can be inferred from any factor. The results of this study showed that insomnia is closely related to mental health, and that insomnia, anxiety, and depression were very prevalent during the pandemic. With that, we emphasize the importance of emotional stability and psychological counseling to reduce the incidence of sleep diseases.

There are several limitations of this study: (i) the sample size is limited and does not fully represent the German public; (ii) the survey was conducted online, was voluntary, and was influenced by the use of electronic devices and other tools; and (iii) there was a deviation in the number of samples recovered as well as in the distribution of age and occupation. In the future, researchers should expand the sample size, increase the number of variables in the questionnaire, and carry out follow-up studies. Although many factors have already been found to affect the public’s sleep quality during the pandemic, it is still necessary to conduct this research in different countries and at different timepoints. High-quality randomized controlled trials are highly recommended to verify the efficacy of different intervention methods on improving sleep quality. At present, ICOSS research has been carried out in 14 countries, and the second version of the survey will cover more questions, thereby providing more comprehensive data for comparative research.

## Conclusion

Our study found that in Germany, insomnia, anxiety, and depression were very prevalent during the pandemic time. Anxiety and depression in the insomnia group were more severe than in the non-insomnia group. Moreover, it was also observed that insomnia and mental health are associated with gender, ethnicity, and employment status. Therefore, medical intervention is warranted to decrease the risks of mental health disorders during the pandemic.

## Abbreviations

BMI: Body mass index
COVID-19: Coronavirus disease 2019
GAD‑2: Generalized Anxiety Disorder two-item score
ICOSS: International COVID-19 Sleep Study
ISI: Insomnia Severity Index
PHQ: The Patient Health Questionnaire for Depression and Anxiety
RKI: Robert Koch Institute
WHO‑5: World Health Organization-Five Well-Being Index

## Declarations

The study was conducted in accordance with the Declaration of Helsinki and approved by the local ethics committee of the Charite University Medicine Berlin (reference number EA1/161/20). All participants gave informed consent prior to the survey.
