# Erratum to: Analysis of the correlations between insomnia and mental health during the COVID-19 pandemic in Germany

**DOI:** 10.1007/s11818-022-00360-w

**Published:** 2022-07-11

**Authors:** Ying Huang, Ingo Fietze, Thomas Penzel

**Affiliations:** Interdisciplinary Sleep Medicine Center, Charité—Universitätsmedizin Berlin, Chariteplatz 1, 10117 Berlin, Germany

**Erratum to:**

**Somnologie 2022**

10.1007/s11818-022-00347-7

In this article the author name Thomas Penzel was incorrectly written as Thmoas Penzel. The original article has been corrected.

